# Equine Ulcerative Genital Lesions Associated with Parapoxvirus

**DOI:** 10.3390/v18080852

**Published:** 2026-08-04

**Authors:** Ann Cullinane, Marie Garvey, Donald Collins, Rachel Lyons, Maura Nelly, Adam Grimes

**Affiliations:** 1 Virology Unit, Irish Equine Centre, Johnstown, Naas, County Kildare, W91 RH93, Ireland; mgarvey@irishequinecentre.ie (M.G.); rlyons@irishequinecentre.ie (R.L.); mnelly@irishequinecentre.ie (M.N.); agrimes@irishequinecentre.ie (A.G.); 2 The Phoenix Equine Ltd., Grey Abbey Road, Kildare Town, County Kildare, R51 YW50, Ireland; doncollins@phoenixequine.com

**Keywords:** parapoxvirus, metagenomics, PCR, horse, equine, genital, anal, lesions, fomite, venereal

## Abstract

In 2026, a widespread outbreak of ulcerative dermatitis of unknown aetiology occurred in breeding horses in Ireland. The lesions resembled those associated with equine herpesvirus 3 (EHV3) infection, i.e., papules, vesicles, and ulcers on the penis of stallions and on the vulval and anal areas of mares. This study aimed to identify the probable causative agent. The methods applied included metagenomic sequencing of DNA, phylogenetic analysis, and real-time PCR. Metagenomic analysis directly from a penile sample confirmed the presence of equine parapoxvirus (EqPPV) DNA. Phylogenetic analysis indicated that it clustered closely with EqPPV first identified in Finland in 2013 from a horse with proliferative dermatitis, and associated with outbreaks of pastern dermatitis in trotting racehorses in 2021/2022. Comparison of the amino acid sequences of the DNA polymerase gene showed that the EqPPVs from Finland and Ireland share 99% identity in contrast to 76–80% with other members of the parapoxvirus genus. A specific PCR test for EqPPV, adapted from that developed in Finland, successfully identified viral DNA in samples from 22 of 25 suspect cases that tested negative for EHV3. In conclusion, this study is the first documented outbreak of genital lesions in breeding horses associated with EqPPV.

## 1. Introduction

Poxviruses are large, complex double-stranded DNA viruses originally named for their association with lesions on the skin. The family, Poxviridae, consists of two subfamilies, Chordopoxvirinae (vertebrate poxviruses) and Entomopoxvirinae (insect poxviruses), with over 20 genera based on host, disease, lack of extensive inter-genus antigenic cross-reactivity, and phylogenetic analyses [1]. The Orthopoxvirus genus of the Chordopoxvirinae includes variola virus (smallpox virus), Mpox virus (previously “monkeypox” virus), cowpox virus, camelpox virus, and horsepox virus. Outbreaks due to horsepox virus are rare to the point of being considered extinct, although multiple clinical forms have been described. These include contagious pustular stomatitis where the lesions are localised to the muzzle and buccal cavity [2], exudative dermatitis of the pasterns (“greasy heal”) [3] and a generalised papular dermatitis [4]. The clinical findings in a Mongolian outbreak in 1976 included pyrexia, pustular stomatitis, lesions on udders and ears, and especially severe disease in foals and mares resulting in several fatalities [5]. The genome sequence of the virus isolated in this outbreak was determined [5], but the identity of many poxvirus infections of horses, often called horsepox, is sometimes uncertain [2].

The Parapoxvirus genus includes ORF virus that primarily infects sheep and goats, bovine papular stomatitis virus, pseudocowpox virus, and red deer parapoxvirus. These viruses are significantly genetically and antigenically different from the orthopoxviruses [1]. Parapoxviruses replicate in epidermal keratinocytes and lesions typically occur around the mouth or teats of mammals. In horses, an infection caused by a parapox-like virus was first verified from a two-year-old standardbred stallion euthanized in Finland in 2013 [6]. The horse exhibited generalised granulomatous inflammation and severe proliferative dermatitis. Other clinical signs included pyrexia, ventral oedema, weight loss and enlarged lymph nodes [6]. Several parapoxviruses are zoonotic, and sequence analysis indicated that the virus from this equine case was closely related to a virus associated with human cases in the USA [6,7]. The virus reemerged in Finland in 2021/2022 when it caused a countrywide epidemic of pastern dermatitis in hundreds of trotting racehorses [8,9]. Sweden reported its first cases of parapoxvirus-associated skin lesions in 2025 [10], but to the best of our knowledge, the virus has not been reported elsewhere to date. This report describes the first documented widespread outbreak of genital and anal lesions in breeding horses associated with equine parapoxvirus (EqPPV).

## 2. Materials and Methods

### 2.1. Samples

Swabs were collected by veterinary clinicians from vesicular, pustular or ulcerated lesions on the external genitalia or anal region of mares, and the penis of stallions. Twenty-eight swabs from 27 horses on nine stud farms were submitted to the Virology Unit at the Irish Equine Centre for routine diagnostics during a six-week period from the end of April to mid-June 2026. Of these 28 swabs, five were from stallions, and the remainder were from mares. The majority of swabs were in virus transport medium (VTM) consisting of phosphate-buffered saline (PBS), 5000 U/mL penicillin, 250 mg/mL amphotericin B and 2% *v*/*v* foetal bovine serum. Dry swabs were processed by adding 5 mL of VTM followed by incubation for a minimum of five minutes. All swab samples were processed using sterile forceps and centrifuged at 526× *g* for 5 min. Samples were stored at 1 °C to 8 °C prior to aliquoting for testing, after which they were archived at −56 °C to −80 °C.

Twenty four genital swabs, five submitted in 2019, nine submitted in 2020, three in 2021, two in 2022, two in 2024 and three in 2025, were retrieved from the Centre’s archives and included as a control group in the investigation.

### 2.2. DNA Extraction

DNA was extracted from 200 µL of supernatant using the Kingfisher Flex Magnetic Particle Processor instrument (Thermo Scientific, Waltham, MA, USA) with the simplex workflow for the MagMax Core Nucleic Acid Purification kit (Cat No: A32702, Applied Biosystems by Thermo Fisher Scientific) as per the kit manufacturer’s guidelines. An exogenous RNA control (Ref: Z-INT-RNA-VIC Primer Design Ltd., Chandler’s Ford, UK) was included in the extraction process.

### 2.3. PCR for Detection of Equine Herpesvirus 3 (EHV3)

Swabs were initially tested for the presence of EHV3 using an in-house real-time PCR assay targeting envelope glycoprotein G (EHV3 gG Forward Primer- TTCAGCCCGGCATATACGA, EHV3 gG Reverse Primer -GGCCCAAACCGGAGGTT, and Probe-6-FAM-AGCGGCGACTTCA-MGB). The assay was performed using the TaqMan 7500 Real-Time PCR system with the AgPath-ID™ One-Step RT-PCR kit (Applied Biosystems by Thermofisher, Austin, TX, USA) and internal control primer/probe mix (Z-INT-RNA-VIC Primer Design Ltd., Chandler’s Ford, UK). The cycling conditions were as follows: 45 °C (10 min), initial denaturation at 95 °C (10 min), followed by 40 cycles of denaturation at 95 °C (15 s) and annealing at 60 °C (45 s, data collection). EHV3 positive and negative controls were included in each run.

### 2.4. Shotgun Metagenomic Sequencing of DNA

A penile sample was prepared for DNA extraction. Six aliquots of 100 µL were taken from the sample to which 2.5 µL of RNAse A solution (4 mg/ML) was added. The mixture was incubated at 37 °C for 30 min with periodic gentle inversion. The treated samples were recombined into 200 µL aliquots for DNA extraction using the QIAamp DNA mini kit (Cat No: 51304, Qiagen, Hilden, Germany). DNA was eluted in a smaller volume (50 µL) of buffer AE, and the isolated DNA was pooled together (total volume ~150 µL). DNA was shipped on dry ice to Novogene (Cambridge, UK), for library preparation and shotgun metagenomics on the NovaSeq X Plus Series sequencing platform (150 paired-end runs, estimated 15 G raw data per sample). A quality control check was carried out prior to library preparation.

### 2.5. Sequence Assembly

Host sequences were removed by mapping reads against the *Equus caballus* TB-T2T reference genome (NCBI RefSeq accession GCF_041296265.1) [11] using SeqMan NGen (DNASTAR 18.1.1, DNASTAR Inc., Madison, WI, USA). The haploid assembly parameters were set to the default mer size and a minimum match of 90%. The remaining unassembled reads (those not associated with the host) were assembled using the same parameters against the NCBI Viral Genome RefSeq Database and the sequences of the Finnish parapox-like virus isolate F14.1158H [12] (GenBank accession numbers OQ248663–OQ248667; five contigs). Reference-based assembly of the largest contig was carried out, a consensus sequence was derived, and sequence similarity was determined.

### 2.6. Phylogenetic Analysis

Phylogenetic analysis was conducted for the DNA polymerase (ORF025) gene. Representative sequences from the subfamilies Chordopoxvirinae (poxviruses of vertebrates) and Entomopoxvirinae (poxviruses of insects) were retrieved from GenBank. Nucleotide sequences were translated into predicted amino acid sequences, aligned using BioEdit (version 7.2.5.0), and the percentage amino acid similarity between the sequences was calculated. Phylogenetic analysis was inferred by Maximum Likelihood using MEGA7 version 7.0.14 [13]. The model with the lowest Bayesian information criterion (BIC) was chosen as the optimal model to describe the amino acid substitution pattern. The tree topology was verified by performing 200 bootstrap replicates.

### 2.7. Detection of EqPPV in Clinical Samples

An EqPPV-specific real-time PCR assay targeting the myristylated protein (ORF093), adapted from Virtanen et al. (2023) [8], was employed to retrospectively screen samples (*n* = 25) collected from 8 farms between the 30 April and the 11 June 2026 and a control group of samples archived in previous years. The primers used were EqPPV F1 128 (ACGAGTGCACAGACCAGTC) and EqPPV R1 (GAGTTCTCCATCACCAGGCT), and the probe was EqPPV P1 (FAM-AAGTGGCTGTTCTTCAGCCAGGA-MGB). The assay utilised the AgPath-ID™ One-Step RT-PCR kit (Applied Biosystems by Thermofisher, Austin, TX, USA). The reaction component (25 µL) for amplification consisted of 12.5 µL of 2× Buffer, 0.4 µM of each primer, 0.16 µM of probe, 0.6 µL of internal control primer/probe mix (Ref: Z-INT-RNA-VIC Primer Design Ltd., Chandler’s Ford, UK), 1 µL of 25× enzyme, nuclease-free water, and 5 µL template DNA. The PCR platform and cycling conditions were the same as for the detection of EHV3. Negative and positive controls (confirmed by DNA sequencing) were included in each run. Samples with a Ct value of <40 were considered positive.

Ten randomly selected real-time PCR positive samples were also tested using pan-pox universal primers that targeted the RNA polymerase subunit gene [14]. The sequences of the high Gc primers used are as follows: forward primer (CAT CCC CAA GGA GAC CAA CGA G), and reverse primer (TCC TCG TCG CCG TCG AAG TC). The reaction component (50 µL) consisted of 2.5 U GoTaq Hot Start Polymerase (5 U/µL) (catalogue no. M5001, Promega, Madison, WI, USA), 10 µL of 5× GoTaq Flexi Buffer, 4.5 µL of 25 mM MgCl_2_ solution, 0.36 mM dNTP Mixture (Thermo Scientific Cat No: R0192), 0.4 of µM each primer, 1 µL of DMSO, 5 µL template DNA, and nuclease-free water (NFW). The cycling conditions were as follows: initial denaturation at 95 °C (5 min), followed by 40 cycles of denaturation at 94 °C (30 min), annealing at 46 °C (1 min), elongation at 72 °C (1 min), and a final extension step at 72 °C for 5 min. PCR reactions were visualised on a 1.2% agarose gel stained with 0.0003% Sybersafe (Invitrogen, Thermo Fisher Scientific, Waltham, MA, USA). A PCR amplicon was purified using the QIAquick PCR purification kit (Cat No: 28106, Qiagen, Hilden, Germany), as per manufacturer’s instructions. Sanger sequencing was performed by Eurofins Genomics Germany GmbH (Anzinger Str. 7a, 85560 Ebersberg, Germany). Nucleotide sequences (forward and reverse) were assembled using SeqMan Ultra (DNASTAR 18.1.1, DNASTAR Inc., Madison, WI, USA) and BLASTN analysis of the consensus sequence against the NCBI nucleotide database was performed [15].

The specificity of the assay was assessed by testing cultured virus stocks of equine herpesvirus 1 (EHV1), equine herpesvirus 4 (EHV4), EHV 3 and equine arteritis virus (EAV).

## 3. Results

### 3.1. Clinical History and EHV3 PCR

**Figure 1:**
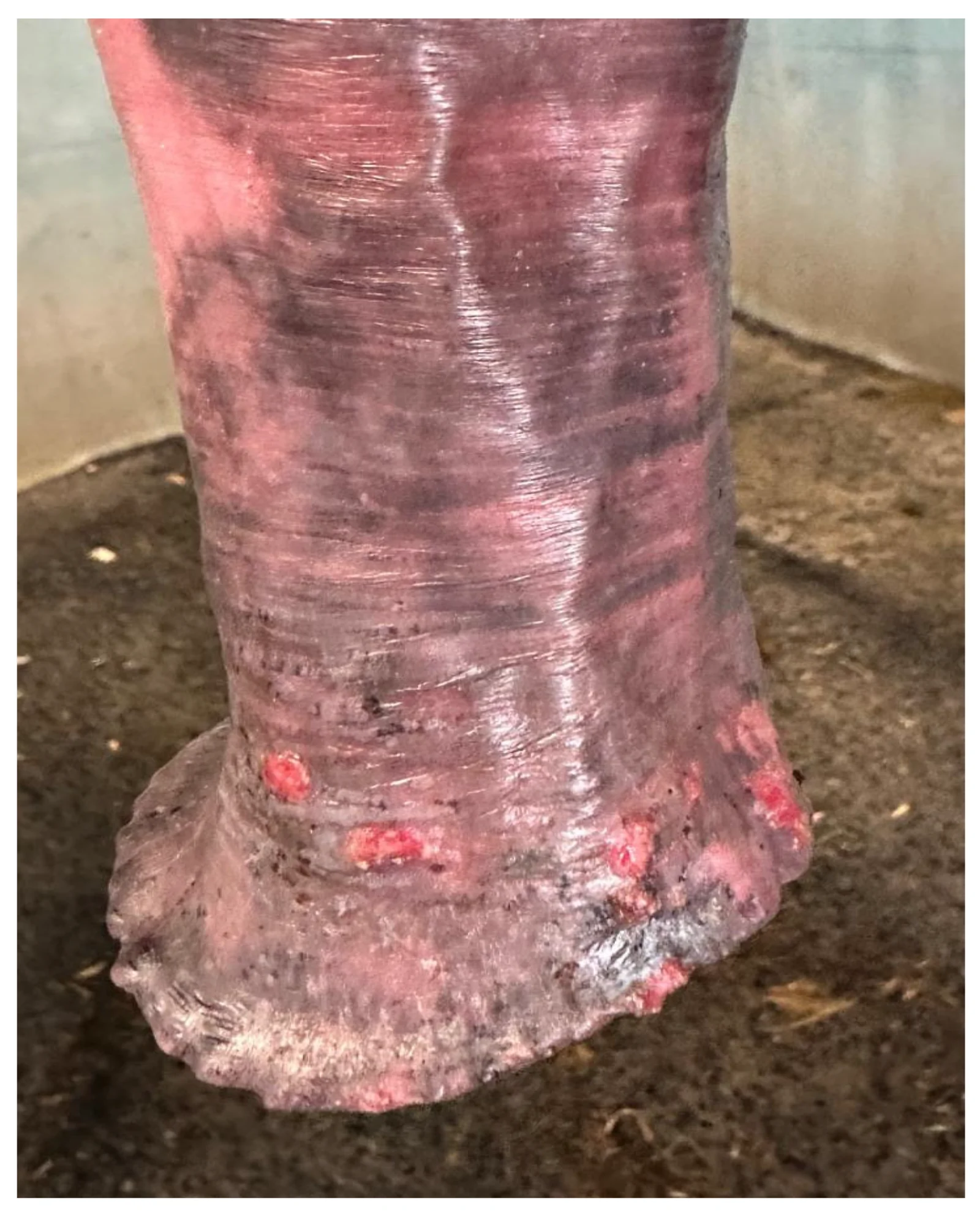
Penile lesions.

**Figure 2:**
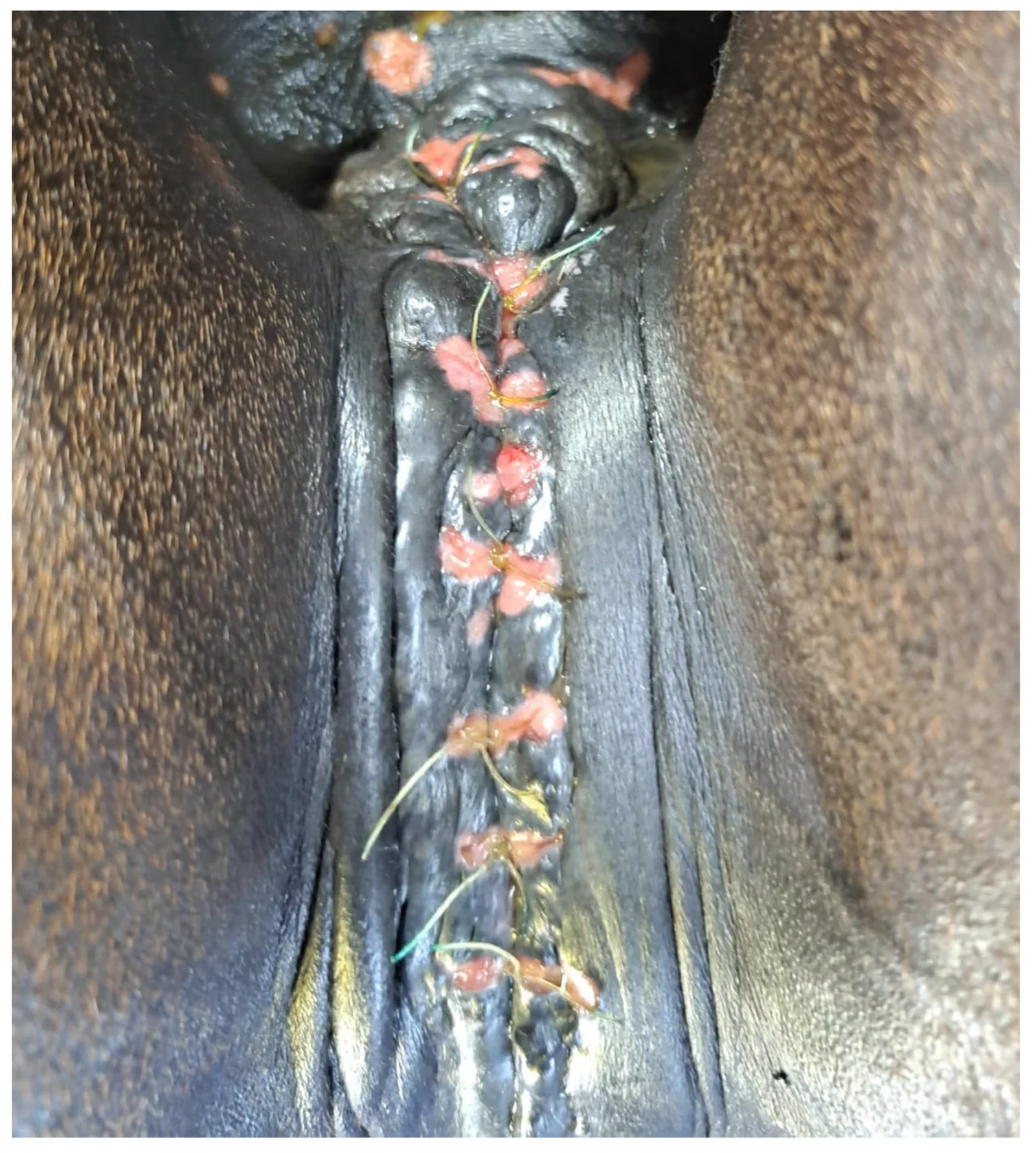
Vulval lesions.

**Figure 3:**
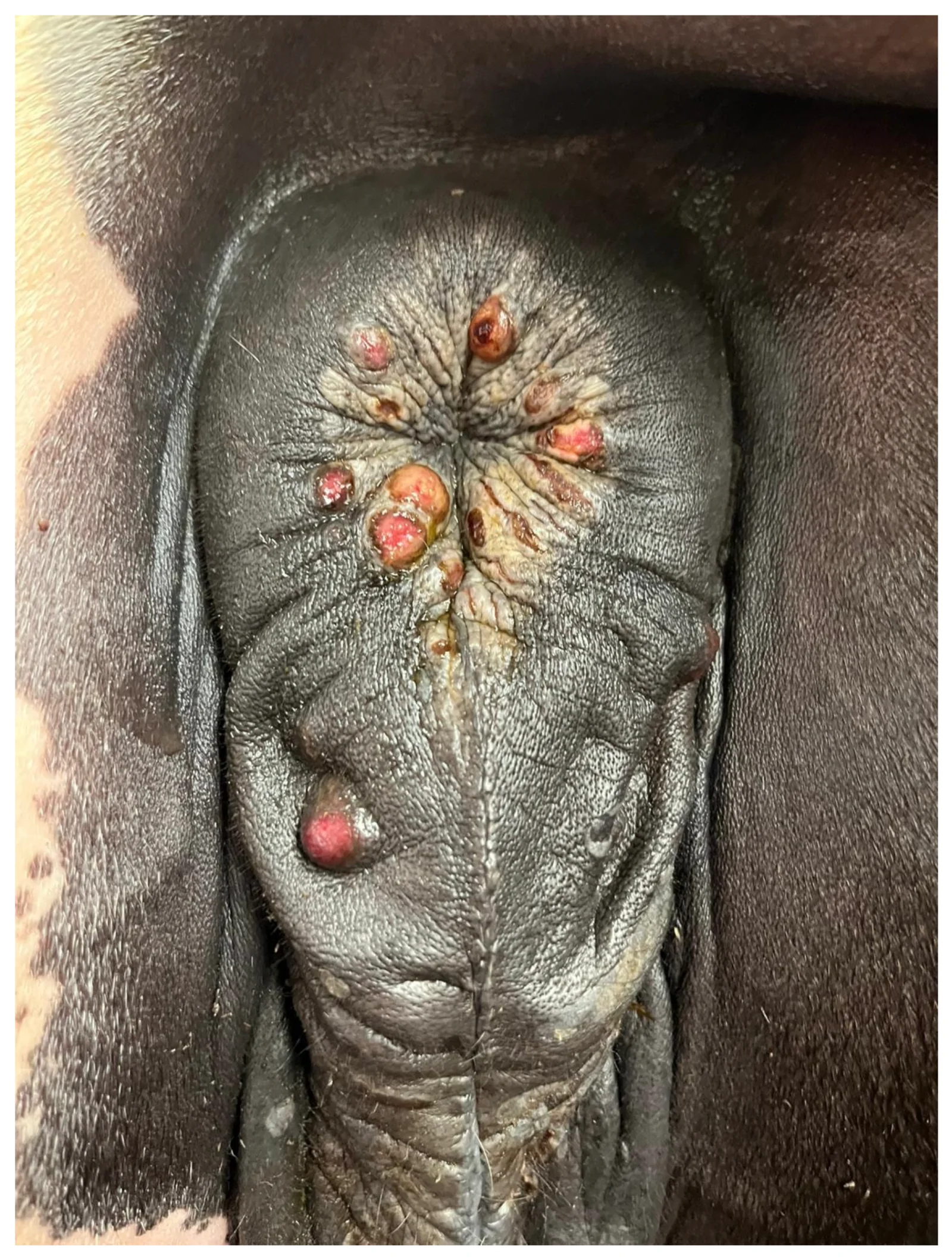
Anal lesions.

From the 30 April to the 5 May 2026, our diagnostic laboratory received samples from two Thoroughbred stallions with ulcerative lesions on the penis. The stallions were on a stud farm where coital exanthema due to equine herpesvirus 3 (EHV3) was confirmed by PCR earlier in the season. Based on clinical presentation and previous history, PCR for EHV3 was requested by the veterinarian, but the virus was not detected. Over the next five weeks, 25 samples from nine premises with horses suffering from the genital lesions highly suggestive of EHV3 infection were submitted to the laboratory. Veterinarians in the field strongly suspected a surge of EHV3 cases, but only two samples on separate premises tested EHV3 positive by PCR. Typically, the lesions described by the clinicians started as erythematous macules that progressed to papules, vesicles, pustules, ulcers, moist crusts, and scars (see Figure 1). Lesions developed simultaneously and were usually multiple but occasionally single. There was variation in the clinical severity, and mares appeared to be more severely affected than stallions (see Figure 2). In some cases, the lesions in mares were concentrated around sutures associated with the Caslick’s procedure or around the anal area (Figure 3). Lesions usually healed within two weeks.

### 3.2. Identification of EqPPV by Metagenomic Sequencing of DNA

Metagenomic sequencing directly from a penile swab generated 107,114,800 sequence reads including 93,634,748 (87.4%) that mapped to the equine genome, and 1,451,005 viral sequences (1.4%). The latter were assembled against the NCBI ReqSeq Viral Genome database and the five contigs of the Finnish equine parapox-like virus isolate F14.1158H. This analysis showed that the greatest number of reads mapped to the Finnish equine parapox-like virus F14.1158H (see Table 1) and to the full genome sequences of the family Poxviridae, genus Parapoxvirus: Pseudocowpox virus (GenBank Accession NC_013804.1: 15,967 sequences), Orf virus (GenBank Accession NC_005336.1: 12,504 sequences), Bovine papular stomatitis virus (GenBank Accession NC_005337.1: 12,686 sequences), Parapoxvirus red deer/HL953 strain HL953 (GenBank Accession NC_025963.1: 12,103 sequences) and the family Papillomaviridae, Dyorhopapillomavirus 1, Equus ferus caballus papillomavirus type 7 (GenBank Accession NC_020501.1: 13,692 sequences) and Dyoiotapapillomavirus 1, Equine papillomavirus 2 (GenBank Accession NC_012123.1: 184 sequences).

Reference-based assembly to the largest contig OQ248663.1 of the poxvirus sequence from the first reported case in Finland in 2013 (Virtanen et al., 2023) [12] showed 100% template coverage with 81,064 sequences mapped to create a single contig of length 66,579 bp. The similarity between the nucleotide sequences was determined as 99.4%, and the Irish virus was subsequently designated EqPPV/IRL/2026/7507.

### 3.3. Phylogenetic Analysis

**Figure 4:**
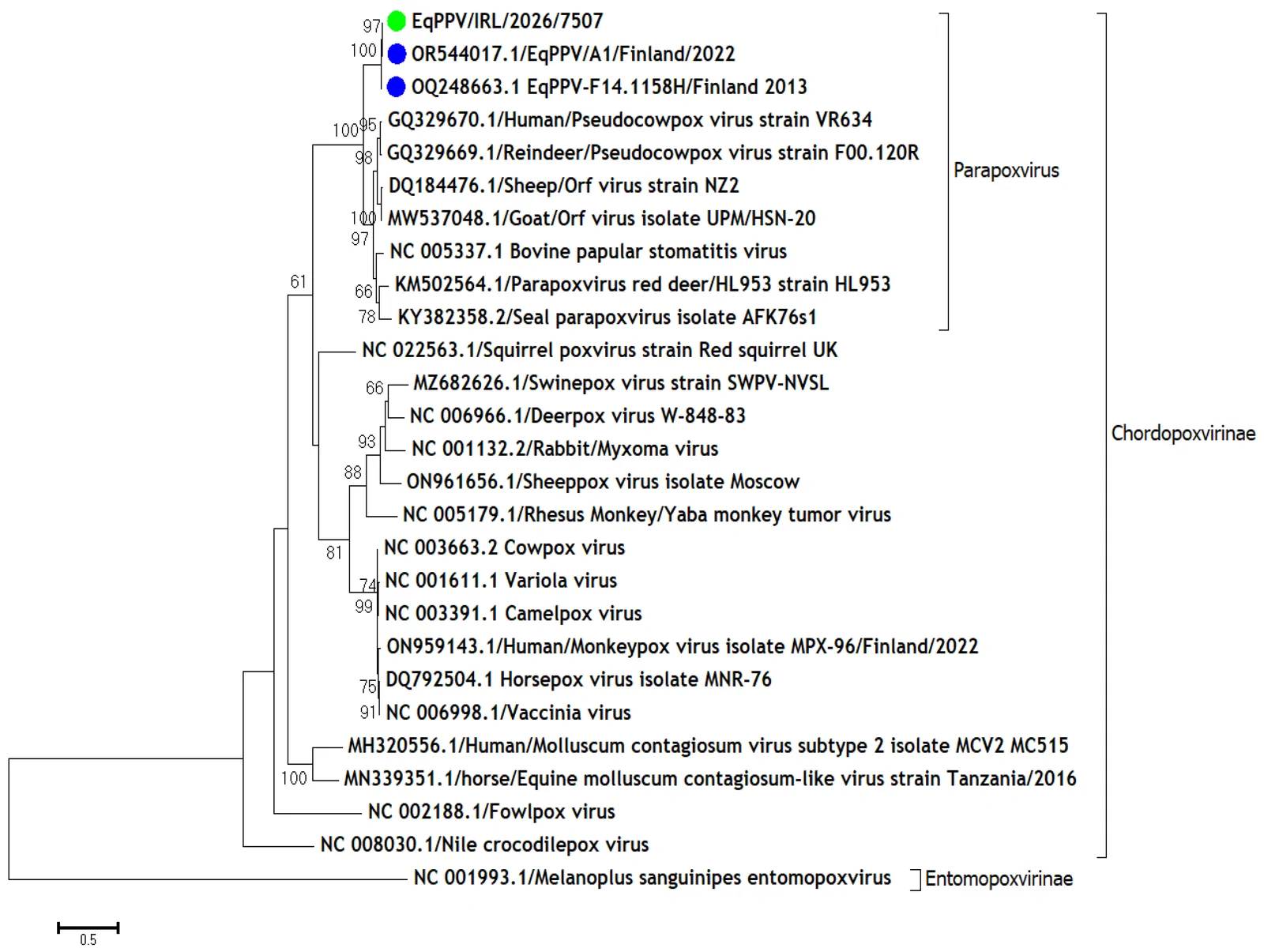
Phylogenetic tree based on amino acid sequences of the DNA polymerase (ORF025) gene of the subfamily Chordopoxvirinae. One strain from subfamily Entomopoxvirinae is used as an outgroup. The Irish EqPPV strain examined in this study is indicated by a green circle, and Finnish EqPPV strains are indicated in blue. Bootstrap values above 60% are shown at each node. Scale bars indicate substitutions per site. GenBank accession numbers are provided for reference sequences.

The evolutionary history was inferred using the Maximum Likelihood method based on the Le_Gascuel_2008 model [16] (lowest BIC score). The tree with the highest log likelihood (−19,930.9649) is shown (Figure 4). Initial tree(s) for the heuristic search were obtained automatically by applying Neighbour-Join and BioNJ algorithms to a matrix of pairwise distances estimated using a JTT model, and then selecting the topology with the superior log likelihood value. A discrete Gamma distribution was used to model evolutionary rate differences among sites (5 categories (+*G*, parameter = 1.7070)) [13].

Within the genus Parapoxvirus, the EqPPVs from Finnish cases in 2013 and 2022 clustered together with the virus characterised from the Irish outbreak in 2026. They form a separate clade from the parapoxviruses of other species. Comparison of the predicted amino acid sequences of DNA polymerase for the three EqPPV viruses included in the phylogenetic analysis showed that they share 99.1–99.9% identity (see Table 2). In contrast, the levels of identity to other members of the genus parapoxvirus are 76.0% to 79.7%.

### 3.4. Parapoxvirus PCR

Twenty-two of 25 clinical samples from suspect cases tested positive by the real-time PCR assay with a Ct range of 18 to 31. All 24 clinical samples submitted in the previous five years tested negative, as did the EHV1, EHV4, EHV3, and EAV controls. Of the 10 real-time PCR-positive samples tested by the less sensitive conventional PCR with the pan-poxvirus primers, five were positive (see Supplementary Figure S1). Confirmatory analysis of the pan-pox amplicon by Sanger sequencing using BLASTn (Standard Nucleotide BLAST) [15] showed 100% identity (606/606 bp) to the Finnish equine parapox isolates (accession numbers: OR610667.1, OQ110632.1, KM491712.1, OR610665.1, OR610666.1, OQ248663.1, KR827441.1).

## 4. Discussion

Metagenomic sequencing has become a very useful tool for identifying emerging or novel pathogens. In Kentucky, it was used to identify a novel equine rotavirus group B as the cause of a major outbreak of diarrhoea in young foals in 2021 [17]. Using metagenomics, this study identified the probable causative agent of a widespread outbreak of ulcerative dermatitis as an equine parapoxvirus (EqPPV) very closely related to the EqPPV associated with a severe outbreak of pastern dermatitis throughout Finland [8,12]. Equine papillomavirus genomes were also identified in the penile swab but were regarded as incidental to the investigation, as papillomaviruses can establish long-term persistent asymptomatic infections and are associated with warts and neoplasia, rather than self-limiting pox-like lesions [18].

Poxvirus genomes are quite stable in evolutionary time, and very little variation in the partial genome sequence was observed between these recent viruses and the sequence from the first case identified in 2013. Comparison of the amino acid sequences of the DNA polymerase (ORF025) of the Finnish and Irish viruses indicated they shared 99% identity. Obviously, this similarity is based on a very limited region of the parapoxvirus genome (typically 130–150 kb), and there may be significant variations in other regions. Determination of the whole-genome sequences of equine parapoxvirus strains has the potential to identify heterogeneity in more variable virulence genes that dictate pathogenicity. In the future, analysing genes with variable sequences could be used to track viruses and elucidate transmission pathways.

Although the viruses were closely related, the epidemiological and clinical findings in Ireland differed from the outbreak in racehorses in Finland. In Ireland, it was breeding stock that was affected, and the lesions, which were similar to those associated with EHV3 infection (papules, vesicles, pustules, and ulcers), were on the penis of stallions and the vulval, perineal, and anal region of mares. Comparable to the Finnish outbreak, there appeared to be widespread geographical spread of disease with clinical cases reported on multiple premises in two of the four provinces of Ireland within a five-week period. To the best of our knowledge, genital and anal infections due to a parapoxvirus have not been described previously in horses but were a feature of the recent Mpox (an orthopoxvirus) outbreak in people where high rates of transmission occurred at sex-on-premises venues [19]. In 2013, an outbreak of parapoxviral vulvovaginitis in first-calved heifers was reported in a dairy farm in California where animals with extensive deep ulcers involving > 50% of vaginal mucosa were euthanised [20]. The mode of transmission was not identified, although direct contact or transmission by personnel and equipment were considered. To date, the possible modes of transmission of equine parapoxvirus are speculative and, in this outbreak, were based on informal discussions with clinicians and not on rigorous epidemiological data. All affected horses were breeding animals on both public and private stud farms, suggesting venereal transmission but also transmission by fomites, possibly on occasion, during rectal examinations of mares as has been reported previously for EHV3 [21]. In some cases, it was reported that the lesions appeared to be more painful than those associated with EHV3 infection. This finding was consistent with the Finnish outbreak of EqPPV associated pastern dermatitis in racehorses when affected horses became lame due to painful lesions [8]. However, unlike the Finnish outbreak, no long-lasting lesions were reported in the outbreak in Ireland. In mares, the disease was frequently first observed at ultrasound scanning for pregnancy at day 14 after ovulation, and in many cases, the lesions had healed by day 28 when a follow-up pregnancy examination was performed.

Many poxviruses have broad host ranges. Human infection with parapoxviruses occurs as a result of direct or indirect (via fomites) contact and usually presents as solitary or regionally restricted self-limiting lesions on the hands or arms. In contrast, immunocompromised individuals may develop lesions in atypical sites such as the face. The confirmation of two human cases infected with a parapoxvirus with a 100% amino acid similarity based on a partial polymerase sequence suggests that this virus or a very closely related virus may pose a threat to people in contact with infected horses or donkeys [7]. In the current outbreak, two suspected human cases (a solitary vesicular lesion on the wrist and a solitary vesicular lesion on a thumb accompanied by lymphadenopathy) were described by veterinarians but not confirmed by laboratory testing. Similarly, skin lesions were reported in people on several affected premises in Finland but without laboratory confirmation [8].

The EqPPV specific PCR test developed by Virtanen et al. (2023) [8] successfully identified the viral DNA in samples from 22 of 25 suspect cases that tested negative for EHV3. In the absence of a vaccine to prevent the EqPPV associated disease, rapid diagnosis and differentiation from the ubiquitous EHV3 will be key to the control of this virus on stud farms in the future. Both viruses are transmitted by direct contact, and infection leads to disruption to breeding activities and economic loss to the Thoroughbred industry, which only permits natural breeding. However, unlike EHV3, which can be alleviated with the topical administration of nucleoside analogues, such as ganciclovir, there are no known effective antivirals against EqPPV in horses [8,22]. Imiquimod has been shown to assist in the clearance of cutaneous poxvirus infections in people but was unsuccessful when used in an immunocompromised patient with facial lesions associated with infection with an EqPPV-like virus [7]. Furthermore, equine herpesviruses are labile enveloped viruses and unlikely to survive in the environment in an infectious form 21 days after depopulation [23], but parapoxviruses can persist for long periods [24]. Both the mature virion and the enveloped virion of poxviruses are infectious [25]. The mature virions primarily mediate host-to-host transmission and are physically resistant to degradation under ambient conditions. When protected in crusts, parapoxviruses, such as the ORF virus, can survive for months or even years in cool, dry conditions [24]. Thus, it is important to thoroughly clean housing, equipment, and handling facilities before disinfecting with an effective product. Sodium hypochlorite is a recommended disinfectant but is rapidly inactivated in the presence of organic matter. In a comparative study, benzalkonium chloride was shown to have similar virucidal activity to sodium hypochlorite and to be more effective in the presence of organic matter [26]. Chlorhexidine is not recommended for treating or disinfecting against parapoxviruses. For topical washing, povidone-iodine is more effective against pox viruses [27]. A recent study during an outbreak in Finland demonstrated the presence of EqPPV DNA throughout the stable environment, including in bedding, bandages, and grooming tools [28]. Shared equipment such as clippers is likely to facilitate indirect transmission.

EqPPV appears to be an emerging threat and serological studies in Finland suggest it may be underdiagnosed, causing disease outbreaks every few years [9]. To date, infection with this virus has manifested as a generalised dermatitis necessitating euthanasia, pastern dermatitis with some horses having long-lasting hoof lesions, and a self-limiting ulcerative dermatitis of external genitalia. Although we are currently unaware of the potential of the virus, we have the tools for rapid confirmatory diagnosis in addition to knowledge extrapolated from studies on disease control measures for other parapoxviruses. Vaccination has proven to be very successful against some pox viruses, and in the absence of a commercial product, consideration could be given to the use of autogenous vaccines. New outbreaks of EqPPV are likely to occur, and it would be beneficial to the horse industry to develop a set of harmonised guidelines to minimise the spread of the disease. These guidelines should include recommendations on effective biosecurity measures, isolation of infected animals, temporary cessation of activities and voluntary movement restrictions to avoid dissemination of virus, disease spread and contamination of other premises.

Limitations of the study include the lack of a negative extraction or sequencing control in the metagenomic analysis of the penile swab. However, the high coverage (81,064 reads) from an index case makes both cross-contamination and environmental contamination in the laboratory unlikely. Furthermore, no parapoxvirus DNA was detected by metagenomic sequencing of samples from horses with other pathologies during the 2026 breeding season. The majority of the reads generated in this study originated from the equine host genome, and of the remainder, only viral sequences were investigated. In Finland, bacteriological results revealed possible secondary infection with pathogenic and zoonotic bacteria [8]. Therefore, future studies will be extended to investigate the microbiota. The real-time PCR assay developed by Virtanen et al. (2023) [8] was successfully deployed in the face of the Irish outbreak to identify positive cases. The specificity of the results was confirmed by other molecular methods such as a pan-pox universal PCR assay, Sanger sequencing and metagenomics, in addition to establishing no false positive results with other viruses and archival samples. However, more rigorous validation, such as the determination of the limit of detection, is required and will be carried out in advance of the next breeding season.

In conclusion, using molecular diagnostics, this study identified EqPPV as the most likely cause of a widespread outbreak of genital and anal lesions in breeding horses. Previously, this virus was associated with a single case of severe proliferative dermatitis and widespread outbreaks of pastern dermatitis in trotting racehorses in Finland. The current findings suggest that infection with EqPPV may result in different clinical outcomes and should be considered in the differential diagnosis of muco-epithelial disease. Real-time PCR is a reliable frontline confirmatory test. Infection may pose a zoonotic risk and biosafety measures need to be implemented to protect those in contact with affected horses.

## Figures and Tables

**Table 1:** Number of sequences mapped to the five contigs of equine parapoxvirus (EqPPV) strain F14.1158H.

GenBank Accession Numbers for Contigs of EqPPV (F14.1158H)	Contig Length (bp)	Sequences Mapped	Percentage Coverage	Median Depth
OQ248663.1	66,579	81,064	100%	295.07
OQ248664.1	11,891	16,536	100%	320.63
OQ248665.1	11,439	14,164	100%	305.47
OQ248666.1	10,770	14,930	100%	324.33
OQ248667.1	21,216	26,841	100%	308.37

**Table 2:** Percentage amino acid identity between the DNA polymerase sequences within the genus parapoxvirus.

	EqPPV/ IRL/2026/7507	EqPPV/A1/Finland/2022	EqPPV/F14.1158H/ Finland 2013	SePPV AFK76s1	RDPV HL953	Goat/Orf Virus UPM/ HSN-20	Sheep/Orf Virus NZ2	Reindeer PCPV/F00.120R	Human/ PCPV/VR634	BPSV/BV-AR02
EqPPV/IRL/2026/7507	100% *	**99.9%**	**99.2%**	76.0%	77.2%	78.8%	78.8%	79.0%	79.7%	77.7%
EqPPV/A1/Finland/2022	**99.9%**	**100%**	**99.1%**	75.9%	77.1%	78.7%	78.7%	78.9%	79.6%	77.6%
EqPPV/F14.1158H/Finland 2013	**99.2%**	**99.1%**	**100%**	76.0%	77.4%	79.0%	79.0%	79.2%	79.9%	77.8%
SePPV AFK76s1	76.0%	75.9%	76.0%	**100%**	84.5%	84.4%	83.8%	84.2%	83.8%	84.8%
RDPV HL953	77.2%	77.1%	77.4%	84.5%	**100%**	85.6%	85.4%	85.2%	85.4%	87.0%
Goat/Orfvirus/UPM/HSN-20	78.8%	78.7%	79.0%	84.4%	85.6%	**100%**	**99.4%**	94.8%	94.3%	87.0%
Sheep/Orf virus/NZ2	78.8%	78.7%	79.0%	83.8%	85.4%	**99.4%**	**100%**	94.5%	94.0%	86.8%
Reindeer/ PCPV/F00.120R	79.0%	78.9%	79.2%	84.2%	85.2%	94.8%	94.5%	**100%**	**98.1%**	87.8%
Human/ PCPV/VR634	79.7%	79.6%	79.9%	83.8%	85.4%	94.3%	94.0%	**98.1%**	**100%**	88.1%
Bovine/BPSV/BV-AR02	77.7%	77.6%	77.8%	84.8%	87.0%	87.0%	86.8%	87.8%	88.1%	**100%**

* Percentage identity greater than 95% is shown in bold. Abbreviations: Equine parapoxvirus (EqPPV), Seal parapoxvirus (SePPV), Parapoxvirus red deer (RDPV), Pseudocowpox virus (PCPV); Bovine papular stomatitis virus (BPSV).

## Data Availability

The raw sequencing data for the equine parapoxvirus (EqPPV) described has been deposited in the NCBI Sequence Read Archive (SRA) under BioProject accession number PRJNA1504398.

## Supplementary Materials

The following supporting information can be downloaded at: https://www.mdpi.com/article/10.3390/v18080852/s1, Figure S1: Agarose gel electrophoresis of samples amplified using High-GC pan-Pox PCR.

## Abbreviations

The following abbreviations are used in this manuscript:

DNA	Deoxyribonucleic acid
EqPPV	Equine parapoxvirus
EHV3	Equine herpesvirus 3
PCR	Polymerase Chain Reaction
NCBI	National Center for Biotechnology Information
VTM	Virus Transport Medium
