# Supplementary material for: Equine Ulcerative Genital Lesions Associated with Parapoxvirus
Source: Viruses. 2026 Aug 4;18(8):852. doi: 10.3390/v18080852 (PMC13517367; doi:10.3390/v18080852)
Supplement: Supplementary file 1 [file viruses-18-00852-s001.zip › viruses-4415993-supplementary.pdf]

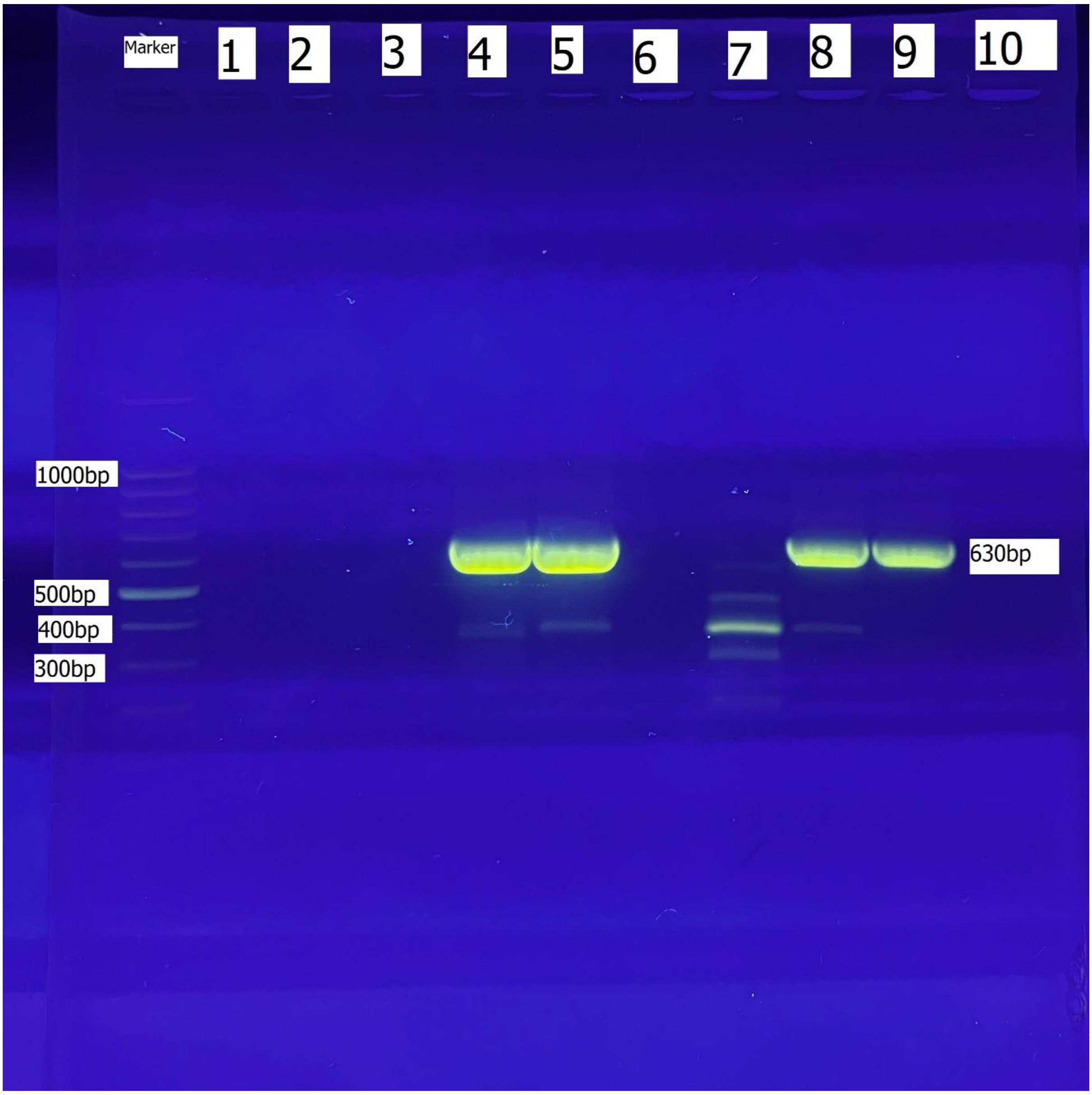

Figure S1. Agarose gel (1.2%) electrophoresis of samples amplified using High-GC pan-Pox PCR [14]. Marker: 100 base pair (bp) DNA ladder. Lane 1: Negative control showing no amplification. Lanes 2, 3, 6, 7, 10: Samples with no amplification. Lanes 4, 5, 8 and 9: positive amplification of the 630bp equine parapoxvirus amplicon.
